# Distortion-product otoacoustic emissions and auditory brainstem responses sensitivity assessment in cisplatin-induced ototoxicity in rats

**DOI:** 10.1016/S1808-8694(15)30483-3

**Published:** 2015-10-19

**Authors:** Marcos Rabelo de Freitas, Viviane Carvalho da Silva, Gerly Anne de Castro Brito, José Valdir de Carvalho, Raimundo Martins Gomes, Ronaldo de Albuquerque Ribeiro

**Affiliations:** 1 PhD, Adjunct Professor of Otorhinolaryngology – Department of Surgery – Medical School – Federal University of Ceará; 2 Assistant Physician – Otorhinolaryngology Ward – Walter Cantídio University Hospital – Medical School – Federal University of Ceará; 3 Associate Professor – Department of Morphology – Medical School – Federal University of Ceará; 4 Medical Student – Surgery Department – Medical School – Federal University of Ceará; 5 Medical Student – Surgery Department – Medical School – Federal University of Ceará; 6 Associate Professor – Department of Physiology and Pharmacology – Medical Student – Surgery Department – Medical School – Federal University of Ceará

**Keywords:** hearing, cisplatin, hearing loss

## Abstract

Cisplatin (cis-diamminedicloroplatinum) is an antineoplastic drug used in the treatment of a variety of cancers, especially head-and-neck cancer. Its ototoxicity, however, has been noted as a common side-effect which limits its use and causes significant morbidity.

**Aim:**

to assess distortion-product otoacoustic emissions (DPOAE) and brainstem evoked response audiometry (BERA) sensitivity to detect secondary ototoxicity caused by different doses and means of administration of cisplatin in rats.

**Study Design:**

Experimental.

**Materials and Methods:**

Male Wistar rats were intraperitoneally (i.p.) injected with 24 mg/kg cisplatin, divided into three equal doses (8mg/kg) or a single i.p. injection of 16 mg/kg. The animals were evaluated by distortion product otoacoustic emission (DPOAE) or brainstem evoked response audiometry (BERA) on the 3rd and 4th days after the cisplatin injection.

**Results:**

Treatment with cisplatin 24 mg/kg resulted in significant DPOAE decrease and it raised the BERA electrophysiological threshold. The 16mg/kg dose could not significantly reduce the DPOAE amplitude, but it raised the animals' hearing thresholds – detected by the BERA.

**Conclusion:**

In rats, BERA was more sensitivity than DPOAE at detecting cisplatin-induced ototoxicity in rats considering different doses and means of administration.

## INTRODUCTION

Cisplatin (cis-diamine-dichloroplatinum – CDDP) is a chemothepeutic drug frequently used to treat many types of cancer, especially those in the head and neck^1^. Its side effects include ototoxicity, nephrotoxicity, medullary suppression and GI disorders^2^. These types of toxicity can interfere with treatment as it reduces treatment dosage, frequence and duration in many patients^3^.

The incidence of cisplatin toxicity can vary between 4 and 50%^4^, ^5^, ^6^. The degree of hearing loss depends on the dose and frequency of drug administration. Bolus doses in adults cause more intense ototoxic damage^7^. Other factors which influence the degree of hearing loss include age (children and the elderly are more susceptible), more chemotherapeutic cycles, previous use of other drugs such as furosemide and ethacrynic acid, associated renal dysfunction and individual susceptibility^8^,^9^. Moreover, because of cisplatin buildup in the body along treatment years, a progressive hearing loss can remain even after treatment suspension^10^.

Animal studies have shown that cisplatin causes degeneration to the organ of Corti, with complete or partial loss of outer hair cells and, sporadically, that of inner hair cells ^11^,^12^. Adding to the ototoxic effects of cisplatin on the organ of Corti, there is evidence of stria vascularis^12^, ^13^, ^14^, ^15^, ^16^, ^17^, ^18^, ^19^, ^20^, ^21^, ^22^, spiral ganglion^12^, 17, 18, 19,^23^, ^24^ and Reissner vestibular membrane involvement^12^,^25^,^26^.

Different doses and means of administration are used in order to achieve ototoxic effects in rodents. The most commonly used means of administration is intraperitoneal (IP). It is well known that cisplatin's chronic effects are less intense than its acute counterparts^15^.

There are many ways to assess hearing involvement in lab animals, varying from simple methods such as the Preyer's effect^27^, all the way to more sophisticated and expensive methods such as the brainstem evoked auditory potentials^28^, ^29^, ^30^, electrococleography^12^,^17^ and evoked otoacoustic emissions^2^,^30^, ^31^, ^32^. Evoked Otoacoustic Emissions (EOA) and brainstem evoked auditory potentials (BEAP), with broad use in clinical practice, are the ultimately more often employed methods to study cisplatin-induced ototoxicity in rodents.

The goal of the present investigation was to assess distortion product evoked otoacoustic emissions (DPEOAE) and brainstem evoked auditory potentials (BEAP) in the detection of ototoxicity secondary to different doses and means of administering cisplatin in rats.

## MATERIALS AND METHODS

We used male Wistar rats weighing between 200 and 348g, kept in cages with free access to food and water, natural cycles of sleep and awake periods, and handled according to the standards advocated by the Brazilian College of Experimentation with Animals (COBEA), which can be found at www.cobea.org.br. This project was submitted to and approved by the Ethics in Research with Small Animals Committee (CEPA) and was approved under protocol number 28/05.

Animals with signs of external ear disorders, such as: external acoustic meatus hyperemia and edema, tumors or impacted ear wax; animals with middle ear disorders such as tympanic membrane opacification, bulging and hyperemia or perforations; animals without distortion product otoacoustic emissions in any of the frequencies investigated (3, 4, 6 and 8 kHz), before drug injection; animals with electrophysiological potential greater than 10dB NA – seen at the brainstem evoked auditory potential, before drug injection, were all taken off the study.

The drugs used in the study were:

Cisplatin (Cisplatex® -Eurofarma 50 mg. Brazil): Freeze-dried powder for injectable solution. Preparation: 50 mg of the powder diluted in 50 ml of saline solution, making up a final concentration of 1 mg/ml; Saline solution 0.9%; Ketamine (Vetarnacol®- König Pharmaceutics 50mg/ml. Brazil); Xylazine (Kensol®- König Pharmaceutics 20 mg/ml. Brazil).

The animals were broken down into 12 groups, listed as follows; here n means the number of rats in each group:

Group 1 (CDDP 24 D3 EOAPD) (n=11): cisplatintreated rats at the dose of 8 mg/kg/day for 3 consecutive days (a total of 24 mg/kg) and assessed before treatment (DO) and three days after its onset (D3) by means of distortion product evoked otoacoustic emissions (DPEOAE).

Group 2 (C 24 D3 DPEOAE) (n=6): rats treated with saline solution at the dose of 8 ml/kg/day for 3 consecutive days (total of 24 ml/kg) and assessed before treatment (D0) and three days (D3) after its onset by distortion product evoked otoacoustic emissions (DPEOA).

Group 3 (CDDP 24 D4 DPEOAE) (n=8): rats treated with cisplatin at the dose of 8 mg/kg/day for three consecutive days (total of 24 mg/kg) and assessed before treatment (D0) and four days (D4) after its onset by distortion product evoked otoacoustic emissions (DPEOAE).

Group 4 (C 24 D4 DPEOAE) (n=6): rats treated with saline solution at the dose of 8 ml/kg/day for 3 consecutive days (total of 24 ml/kg) and assessed before treatment (D0) and on the 4th day after its onset (D4) distortion product evoked otoacoustic emissions (DPEOAE).

Group 5 (CDDP 16 D3 DPEOAE) (n=12): rats treated with cisplatin in a single dose of 16 mg/kg/day, assessed before injection (D0) and three days after (D3) its onset by distortion product evoked otoacoustic emissions (DPEOAE).

Group 6 (C 16 D3 DPEOAE) (n=5): rats treated with saline solution at a single dose of 16 ml/kg/day, assessed before treatment (D0) and three days (D3) after its onset by distortion product evoked otoacoustic emissions (DPEOAE).

Group 7 (CDDP 16 D4 DPEOAE) (n=7): rats injected with cisplatin in a single dose of 16 mg/kg/day, assessed before treatment (D0) and four days (D4) after its onset by distortion product evoked otoacoustic emissions (DPEOAE).

Group 8 (C 16 D4 DPEOAE) (n=6): rats injected with saline solution at a single dose of 16 ml/kg/day, assessed before injection (D0) and four days (D4) after its onset by distortion product evoked otoacoustic emissions (DPEOAE).

Group 9 (CDDP 24 BEAP) (n=11): rats injected with cisplatin at the dose of 8 mg/kg/day for 3 consecutive days (total of 24 mg/kg) and assessed before treatment (D0) and three (D3) and four (D4) days after its onset by brainstem evoked auditory potentials (BEAP).

Group 10 (C 24 BEAP) (n=7): rats injected with saline solution at the dose of 8 ml/kg/day for 3 consecutive days (total of 24 ml/kg) an assessed before the injection (D0), three (D3) and four (D4) days after its onset by brainstem evoked auditory potentials (BEAP).

Group 11 (CDDP 16 BEAP) (n=12): rats injected with a single 16mg/kg/day dose of cisplatin and assessed before treatment (D0), three (D3) and four (D4) days after its onset by means of brainstem evoked auditory potentials (BEAP).

Group 12 (C 16 BEAP) (n=8): rats injected with a single 16ml/kg/day dose of saline solution and assessed before injection (D0), three (D3) and four (D4) days after its onset by brainstem evoked auditory potentials (BEAP).

The entire procedure is based on two experiments:

In experiment 1, Wistar rats were submitted to profound anesthesia with 50mg/kg ketamine plus 10mg/kg xylazine. Their ears were previously examined through otoscopy in order to take off the study those animals with signs of middle or external ear disorders, as per described in the exclusion criteria. Those animals with normal otoscopy were submitted to distortion product evoked otoacoustic emissions (DPEOAE), immediately before drug administration. In the 8ml/kg cisplatin for 3 consecutive days and 8 ml/kg for 3 days of saline solution groups, the drugs were intraperitoneally injected immediately after the auditory evaluation. In the two subsequent days, after weighing the rats again, they were again injected with 8mg/kg of cisplatin or 8ml/kg of saline solution, in order to result in a final dose of 24mg/kg and 24ml/kg, respectively. Twenty four (D3) or 48h (D4) after the last injection, the rats were once again anesthetized and another otoscopy was carried out in order to exclude those which had acquired middle or external ear disorders during the drug administration period, and were then submitted to a new auditory evaluation by means of DPEOAE. In the groups which received 16mg/kg of intraperitoneal cisplatin and 16ml/kg of saline solution, we used a Kd Scientific Series 100 infusion pump in order to fix the infusion time in 30 minutes. When necessary, a new dose of anesthetics was used. Hearing assessment was also carried out on the third (D3) and fourth (4D) days after drug injection.

In experiment 2, the animals were submitted to the same anesthesia, otoscopy and drug injection procedures reported for the first experiment. Auditory assessment was carried out by means of a BEAP (Brainstem Evoked Auditory Potential), and the same group of animals was submitted to testing immediately before and on the third and fourth days after drug administration.

DPEOAE were captured by the MADSEN Capella – GN Otometrics Otoacoustic Emissions device in a silent room. The rats were anesthetized and a probe was coupled to their right external auditory canal (probes used in newborn babies). The stimulus was made up of 2 pure sounds (F1 and F2), which F1/F2 frequency ratio was 1.22. Stimuli intensity was fixed in 70 dB SPL. We analyzed a total of 1000 stimuli. The resulting otoacoustic emissions were assessed in the frequencies of 3, 4, 6 and 8 kHz. DPEOAE present were considered when we achieved a signal/noise ratio of at least 6 dB SPL, according to the technical specifications on the device used.

In order to perform the BEAP, we used the EP 25 device from Interacoustic, in a silent environment. With the animals properly anesthetized, platinum subdermal electrodes were placed on the vertex (positive), right retroauricular region (negative) and on the nasal tip (ground). ER-3A insertion phones were coupled to a probe (used to assess the hearing of newborns) and introduced into the rat's external right auditory canal. The stimuli used were rarefaction clicks, released at a rate of 15 per second, with a maximum of 700 stimulations and an analysis time of 15msec. The passing band used was from 0 to 3,000 Hz. The stimuli started at 80 dB HL and progressively reduced until the waves totally disappeared. For the electrophysiological hearing threshold, we considered the lower stimulus intensity in which wave II could be noticed.

For graph creation and statistical analysis we used the GraphPad Prism 4.00.255 software. We assessed the sample distribution by means of the Komogorov-Smirnov methods. Results were expressed as mean ± mean standard error (MED ± PM), for continuous data and as median (Md) and minimum (Min) and maximum (Max) values for the original data. The minimum significance accepted was 5%. The many experimental procedures were compared using the following tests:

Student t test (when possible, to be used with pairing): to compare the means of the distortion product otoacoustic emission for each frequency before and after treatment; compare the mean values of the electrophysiological thresholds of the animals obtained through the brainstem evoked auditory potential between the first (D0) and the fourth (D3) days, for the control (saline) and 24mg/kg for the cisplatin group.

The ANOVA variance analysis with Intergroup Significance established by the Tukey test: to compare the mean values of the electrophysiological thresholds of the animals obtained by means of the brainstem evoked auditory potential between the first (D0), fourth (D3) and the fifth (D4) days of assessment; compare the mean I-V interval values obtained through the brainstem evoked auditory potential between the first (D0), fourth (D3) and the fifth (D4) days of assessment.

## RESULTS

Cisplatin-induced ototoxicity was confirmed by light microscopy under hematoxylin-eosin (HE) (paper submitted to publication).

Group 1 (CDDP 24 D3 DPEOAE) X Group 2 (C 24 D3 DPEOAE).

**Figure 1:**
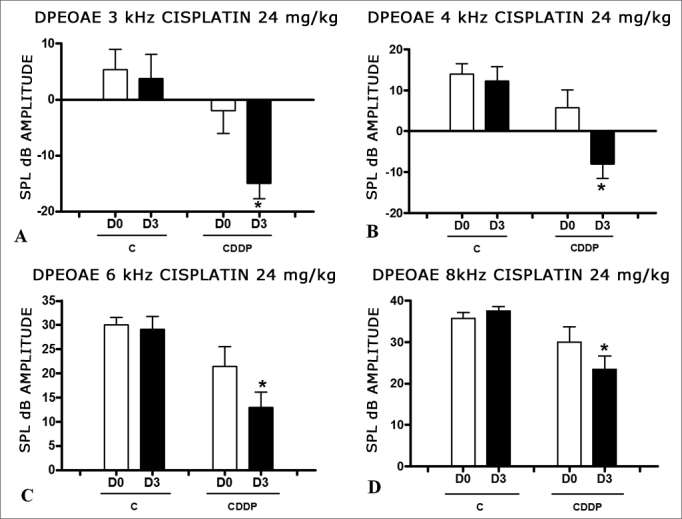
Graph of the DPEOAE amplitude expressed as mean and standard error in the average of days D0 and D3, in groups 1 and 2. The asterisks represent statistical significance. CDDP = cisplatin; C = control. Graph A: 3 kHz frequency (Test T: * p = 0.0095 – D3 × D0 CDDP). Graph B: 4 kHz frequency (Test T: * p = 0.0073 – D3 × D0 CDDP). Graph C: 6 kHz frequency (Test T: * p = 0.0284 – D3 × D0 CDDP). Graph D: 8 kHz frequency (Test T: * p = 0.0338 – D3 × D0 CDDP).

There was a significant reduction in DPEOAE amplitudes in the frequencies tested (3, 4, 6 and 8 kHz) between days D0 and D3, in the group that received CDDP at the dose of 24 mg/kg (Group 1). Such reduction was not seen in the control group. (Figs. 1 A, B, C and D).

Group 3 (CDDP 24 D4 DPEOAE) X Group 4 (C 24 D4 DPEOAE)

**Figure 2:**
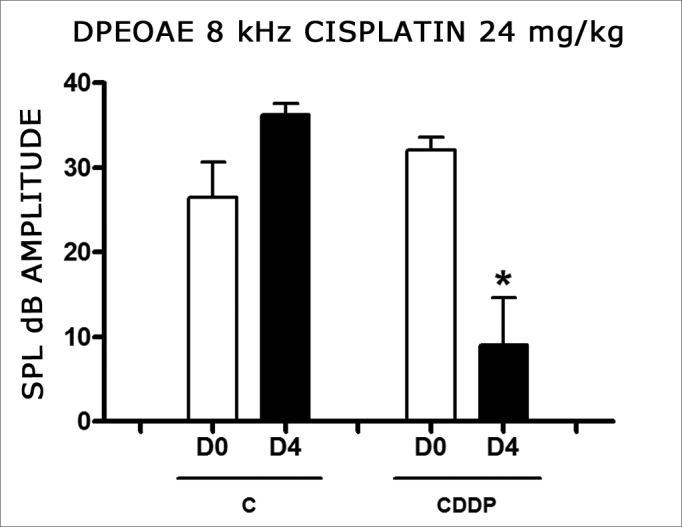
Graph showing DPEOAE amplitudes expressed as mean and error deviation of the average values in days D0 and D4, in groups 3 and 4 at the frequency of 8 kHz. The asterisk represents statistical significance. CDDP = cisplatin; C = control. T Test: * p = 0.0459 (D4 × D0 CDDP).

In group 3, there was a high mortality among the animals assessed on D4 of the procedure. Of the eight animals which received CDDP at the cumulative dose of 24 mg/kg, only 3 remained alive for DPEOAE on D4. Of these, 2 of 3 rats had their DPEOAE disappearing, proven by a signal/noise difference below 6 dB SPL, in the frequencies of 3 and 4 kHz. One animal also did not show DPEOAE in the frequency of 6 kHz. At the frequency of 8 kHz there was a significant reduction in DPEOAE amplitude between D0 and D4 (Fig. 2).

Group 5 (CDDP 16 D3 DPEOAE) X Group 6 (C 16 D3 DPEOAE)

**Figure 3:**
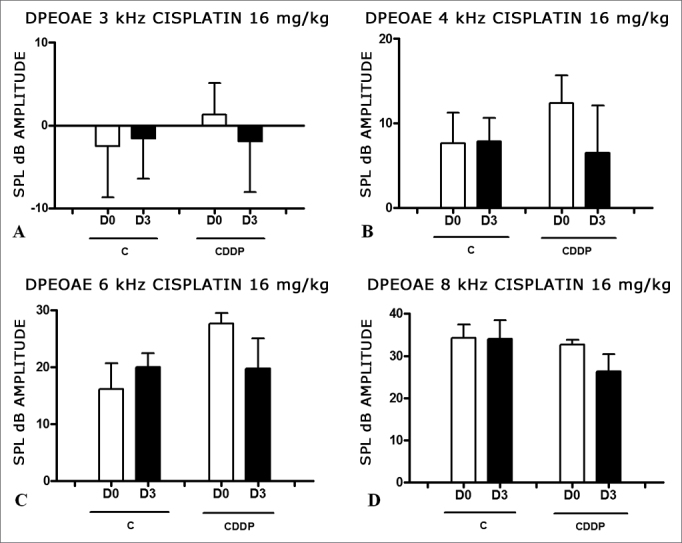
Graph showing DPEOAE amplitudes expressed as mean and error deviation of the average values in days D0 and D3, in groups 5 and 6. CDDP = cisplatin; C = control. There was no statistical meaning in any of the groups (T Test: p > 0.05).

At the dose of 16 mg/kg of CDDP, it was not possible to identify statistically significant reduction in mean DPEOAE amplitudes between D0 and D3, in all the frequencies investigated (p > 0.05) (Figs. 3 A, B, C and D).

Group 7 (CDDP 16 D4 DPEOAE) X Group 8 (C 16 D4 DPEOAE)

**Figure 4:**
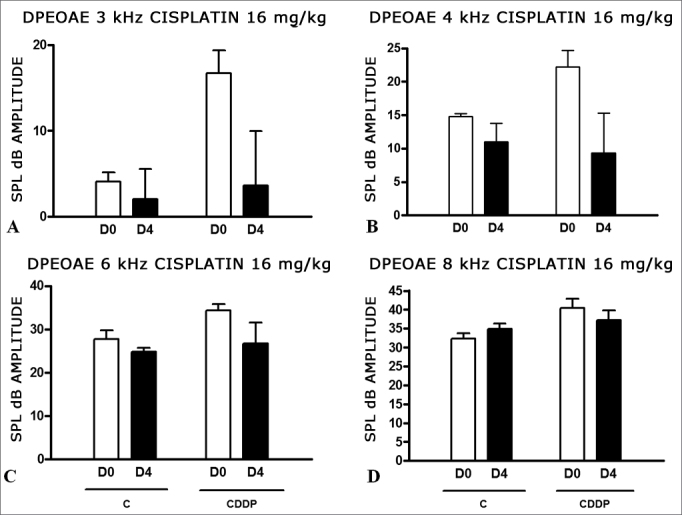
Graph showing DPEOAE amplitudes expressed as mean and error deviation of the average values in days D0 and D4, in groups 7 and 8. CDDP = cisplatin; C = control. There was no statistical meaning in any of the groups (T Test: p > 0.05).

As it happened on the 3 day assessment at the dose of 16 mg/kg of CDDP, it was not possible to identify a statistically significant reduction on the mean amplitudes of DPEOAE between D0 and D4, in all the frequencies investigated (p > 0.05) (Figs. 4 A, B, C and D).

Group 9 (CDDP 24 BEAP) X Group 10 (C 24 BEAP)

**Figure 5:**
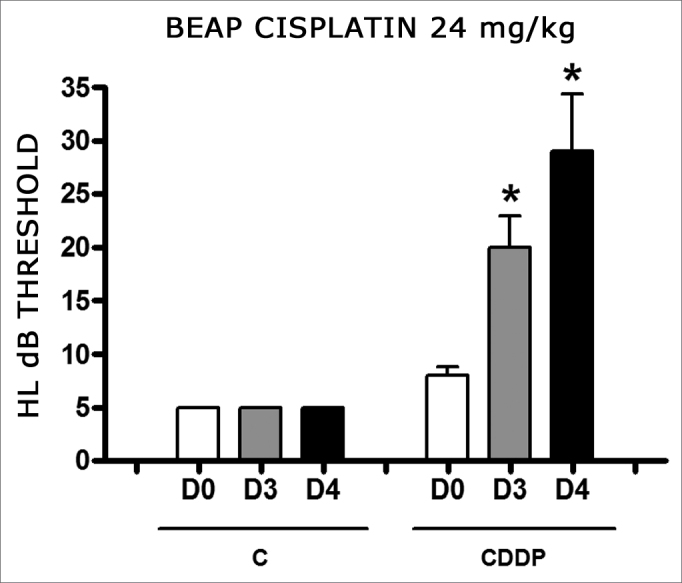
Graph of the mean electrophysiological thresholds in groups 9 and 10 at D0, D3 and D4, expressed as mean and mean standard error (MED and EPM). The asterisk represents a statistically significant difference. CDDP = cisplatin; C = control. ANOVA – Tukey: * p < 0.01 (D3 × D0 CDDP); * p < 0.001 (D4 × D0 CDDP).

**Figure 6:**
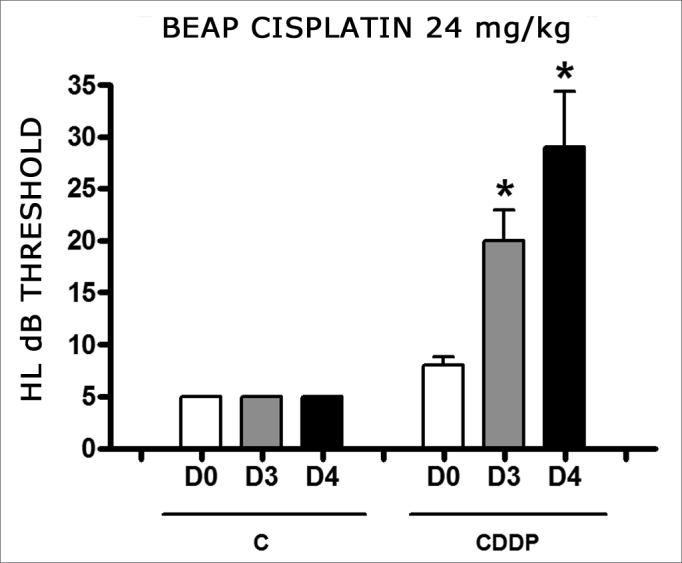
Graph showing the mean I-V interval of the animals in groups 9 and 10 at days D0, D3 and D4 expressed as mean and mean standard error (MED and [IEY1] EPM). We did not find statistical difference between days in the two groups. ANOVA p > 0.05.

There was a significant increase in the mean electrophysiological threshold of animals injected with 24mg/kg of cisplatin on D3 and D4 when compared to D0. We did not notice statistically significant differences considering these thresholds between D3 and D4 when compared to D0. We did not notice statistically significant differences in these thresholds between D3 and D4. In the control group, there was no increase in electrophysiological thresholds (Fig. 5). We did not find statistically significant difference of the I-V interval among the days in both groups (Fig.6).

Group 11 (CDDP 16 BEAP) X Group 12 (C 16 BEAP)

**Figure 7:**
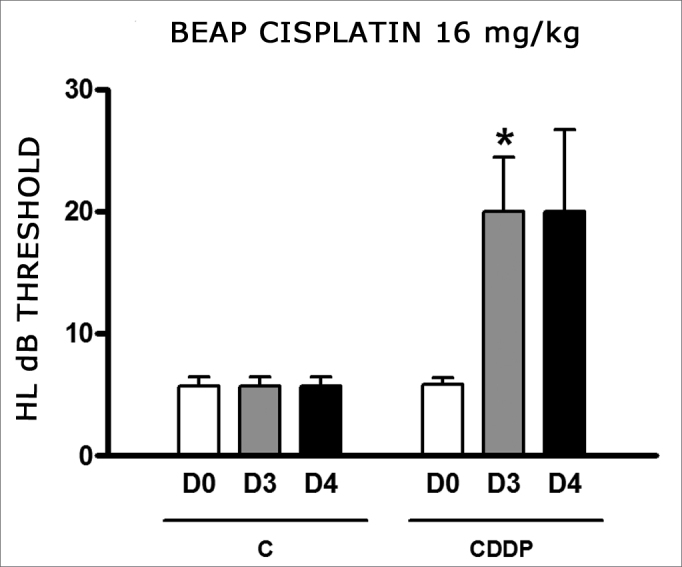
Graph of the mean electrophysiological thresholds in groups 11 and 12 at D0, D3 and D4, expressed as mean and mean standard error (MED and [IEY2] EPM). The asterisk represents a statistically significant difference. CDDP = cisplatin; C = control. ANOVA – Tukey: * p < 0.05 (D3 × D0 CDDP).

**Figure 8:**
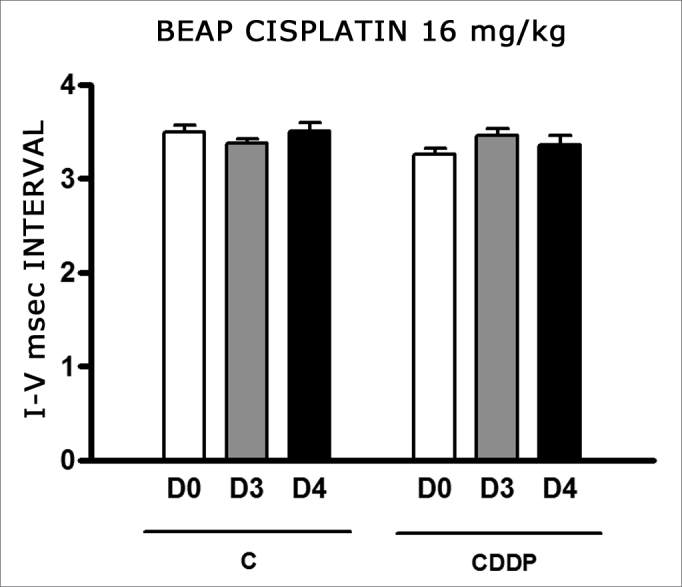
Graph of the mean I-V interval of the animals in groups 11 and 12 at days D0, D3 and D4 expressed as mean and mean standard error (MED and [IEY3]EPM). We did not find statistical difference among the days of the 2 groups. ANOVA p > 0.05.

We found a significant increase in the mean electrophysiological threshold of the animals injected with 16 mg/kg of cisplatin at D3 when compared to D0. We did not notice significant difference in these two thresholds between D3 and D4. In the control group there was also no raise in the electrophysiological thresholds (Fig. 7). We also did not find statistically significant difference in the I-V interval between the days in both groups (Fig. 8).

## DISCUSSION

In this experiment only the right side temporal bone would be used for the different procedures, in order to make more homogeneous the time between cochlear removal and its fixation, thus avoiding damaging the organ to the utmost associated with its removal, which was carried out by the author only. Morphological evaluation data by light microscopy will be published in another paper. The animals submitted to BEAP did not have their cochleas removed, and therefore could be assessed on the third and fourth days after the procedure started. That is why it is necessary to have four groups for BEAP assessments and 8 for DPEOAE.

The intraperitoneal administration route used in this study proved efficient to trigger ototoxicity in the doses used. This is also the systemic administration route seen in most ototoxicity experiments with CDDP in rats. However, other routes can be used such as the arterial^33^, venous^34^, subcutaneous^35^ and intramuscular^15^.

DPEOAE are a hearing assessment method which represents the outer hair cell function, one of the main cisplatin-damaged targets^36^. DPEOAE execution protocol in animal experiments bear little variability, with the greatest controversy happening around the possibility of keeping constant an F1 and F2 stimulus intensity^30^,^32^ or variable^36^, ^37^, for the Dpgram type of acquisition. The possible frequencies to be studied through DPEOAE vary among the species of animals. Hyppolito et al.^32^ identified its presence from 1.5 kHz in guinea pigs; Hatzopoulos et al.^37^, studied them in Sprague-Dawley rats, beyond 4 kHz; Lopez-Gonzalez et al.36, in Wistar rats, identified them between 1 and 6 kHz; Sockalingan et al.^30^, in albino rats, between 2 and 8 kHz for a signal/noise ratio ⩾ 3 dB SPL. McAlpine and Johnstone38 found CDDP ototoxicity through DPEOAE with I/O function shape fixing the frequency in 8 kHz. In the present investigation, we carried out the protocol with a fixed intensity of 70 dB SPL, attaining measurable responses from 3 kHz for a signal/noise ratio ⩾ 6 dB SPL as established by the technical specifications of the device used (Madsen-Capella).

In the present study, only the 24 mg/kg dose was able to trigger a DPEOAE measurable cisplatin-induced ototoxicity in all the frequencies evaluated. With D3 assessment, differently from the control group, there was a significant reduction in DPEOAE amplitude in the treated group. At D4, there was no response or amplitude reduction. In the animals treated with 16 mg/kg, although there was a DPEOAE reduction in the 3, 4, 6 and 8 kHz frequencies after CDDP treatment both in D3 and D4, it was not possible to show statistical significance. This can be explained by the large individual variability in response to ototoxicity by CDDP in rats for doses ⩾ 15 mg/kg in bolus infusion, yielding a greater amplitude standard deviation in otoacoustic emissions in the samples, reducing the statistical tests power. This variability is followed of different levels of plasma concentration of the drug when injected in the vein or peritoneum^37^. Sockalingam et al.^30^ also did not show DPEOAE significant reductions in rats after 72h for the dose of 12 mg/kg of CDDP. Hatzopoulos et al.37 found a significant reduction in the signal/noise ratio (S/N) of DPEOAE for the frequencies of 6,34, 7,13 and 7,56 kH, and it was not seen for 4, 5 or 8 kHz. It is worth stressing that in this last experiment, the parameter assessed was the S/N ratio and not the otoacoustic emissions amplitude alone. Lopez-Gonzalez et al.^36^, used a lower CDDP dose (10 mg/kg), and noticed a significant reduction in DPEOAE in Wistar rats between 1 and 6 kHz as of the seventh day of assessment, returning to initial values after 30 days. Hyppolito et al.^31^, in their study with albino guinea pigs using 24 mg/kg found no DPEOAE in 100% of the animals treated and assessed on the third day after its onset. We did not find rat studies with 24 mg/kg of CDDP. The papers showing DPEOAE reduction after CDDP injection in rats either used a longer assessment time with lower doses^38^ or did not use DPEOAE amplitude as an assessment parameter, but rather the S/N ratio^37^. This type of analysis raises the experiment bias, since the noise detected in the external auditory canal of the animals can be influenced by external factors, such as respiratory noises^39^. Therefore, in the present study, we chose to assess DPEOAE amplitude and not S/N ratio, for a maximum of 96h after treatment, because of the high animal mortality after this time with the doses of 16 or 24 mg/kg.

Thus, in the present investigation, the DPEOAE were able to identify cisplatin-induced ototoxicity in rats only with the 24mg/kg dose.

The brainstem evoked auditory potentials have been broadly used to study cisplatin-induced ototoxicity in rodents^22^, 28, 29, 30, 30, 31, 32, 33, 34, 35, ^37^, 40, 41, 42, 43, 44, 45, 46, 47, 48, 49, 50. However, there is no exam technique standardization among the many authors.

In the present study we could notice a BEAP trace in Wistar rats with the identification of 5 to 7 waves, and wave II had the highest amplitude. For that, we used needle-shaped platinum electrodes, placed on the vertex subdermal (positive or active electrode), right side retroauricular region (reference or negative electrode and nasal tip (ground electrode). The ground electrode may be placed anywhere in the body without altering the trace. Different positions of the reference and active electrodes make some waves more or less evident^51^. Amsallem and Andrieu-Guitrancourt^34^ placed the electrodes between the vertex (active) and tail (reference); Li et al.^52^ between the vertex (positive) and neck muscles (negative). Many authors of the same research group published studies positioning the electrodes at the vertex (positive) and nasal tip (negative)^28^,^29^,^43^, ^45^, ^48^, ^49^,^53^. In other studies, especially the most recent ones, the electrodes were placed at reference points similar to those used in the current experiment^22^, ^33^, ^37^, ^42^, ^46^, ^50^. It is believed that in the clinical records this location be the one that offers an ideal BEAP trace^54^.

There is controversy regarding how to establish the electrophysiological threshold in rats by means of BEAP. In agreement with Amsallem and Andrieu-Guitrancourt^41^, this study showed that wave II is the one with the greatest amplitude in rats and the last one to disappear with the reduction in the sound stimulus intensity. Therefore, this wave was the parameter used to establish the auditory threshold in the animals. Kamimura et al.^28^ also considered wave II in order to establish the electrophysiological threshold. According to Hatzopoulos et al.^42^, wave III is the one with the greatest visibility and reproducibility, thus being the one that establishes the electrophysiological threshold. Tanaka, Whitworth e Rybak^48^ had two replicable peaks as parameters for any wave, while Minami, Sha and Schacht^50^, considered waves III or IV. Others still establish the threshold by means of visually detectable and reproducible traces, without specifying any wave type of quantity^52^,^55^.

The number of stimuli necessary to trigger reproducible BEAP traces varies between 128^30^ and 1024^30^,^33^,^50^ in rat experiments. The rate of stimuli presentation varies between 5 per second ^28^,^29^,^43^, ^44^, ^45^, ^46^, ^48^, ^53^ and 20 per second^30^,^46^. In the present investigation we could see reproducible responses using a stimuli rate of 15 per second for 300 to 700 stimuli.

Anther variable parameter is the type of stimuli used. Most of the authors prefer the tone burst, with or without clicks^22^,^28^,^29^,^33^,^37^,^42^, 44, 45, 46, 47, 48, 49, 50, ^52^, ^53^. The tone burst has the advantage of assessing hearing in a frequency-specific way. Its use is important when one wishes to differentiate whether the lesion affects the cochlear areas responsible for lower or higher frequencies. However, the higher the number of tone bursts used, the longer the test becomes, increasing the mortality risk in animal experiments because of a longer anesthesia time. The click stimulus establishes a physiological response that represents a hearing frequency range between 1000 and 4000 Hz^51^. With that, it triggers a greater nerve fiber depolarization synchronism, making the trace more constant and reproducible than other types of stimuli^56^. Thus, similarly to what happens in other studies^30^, ^34^, ^41^, this experiment was performed using only the rarefaction click as a stimulus.

Although the responses triggered by the click are represented on the cochlear middle turn in rats and not in the basal turn^57^ – a site where the cisplatin-induced lesion prevails^28^,^48^,^50^,^55^,^58^, the 16 e 24 mg/kg doses of cisplatin were able to cause BEAP-measurable ototoxicity. The mean increase in the electrophysiological threshold for the 24 mg/kg dose was of 12 dB HL on D3 and 21 dB HL on D4. However, there was no statistically significant difference between D3 and D4. The 16 mg/kg dose caused a mean threshold increase of 14.17 dB HL both at D3 and D4, with this last day of assessment, having greater threshold variability secondary to increased animal mortality. There was no variation in BEAP threshold in the control group. The mean increase in the electrophysiological threshold for clicks after 16 mg/kg of cisplatin infusion and 72h assessment varied in the literature between 15.2 dB42 and 36 dB44. Sockalingam et al.^30^ did not show a significant threshold increase for clicks after 3 days, with the dose of 12 mg/kg. On the other hand, Tanaka, Whitworth and Rybak48 showed an average increase of 18.3 dB for the dose of 13 mg/kg. We did not find BEAP studies in the hearing assessment of rats for the doses of 24 mg/kg.

Through BEAP there was also a cisplatin-triggered hearing lesion from cochlear structures, since there was no significant increase in the I-V interval in treated animals. A similar result was seen by Rebert, Pryor and Frick^41^. In hamsters, Church et al.^59^ showed I-IV interval stretching with the dose of 15 mg/kg of CDDP, suggesting a retrocochlear lesion for this drug.

Therefore, brainstem evoked auditory potentials (BEAP), using rarefaction clicks as auditory stimuli was an efficient method to detect CDDP ototoxicity in rats in the two doses employed. It is possible that this finding be associated with a great variability (standard deviation) of the DPEOAE amplitudes among the animals investigated.

## CONCLUSIONS

BEAPs were more sensitive than DPEOAE in detecting cisplatin-induced ototoxicity in rats in different doses and means of administration.
